# Association of Minimally Invasive Hysterectomy Route at the Time of Sacrocolpopexy with Postoperative Complications

**DOI:** 10.1007/s00192-025-06175-1

**Published:** 2025-06-11

**Authors:** Selma Su, Sophia Neman, Naomi Fields, Douglas Luchristt, C. Emi Bretschneider

**Affiliations:** 1 Division of Urogynecology and Reconstructive Pelvic Surgery, Northwestern Medicine, Chicago, IL USA; 2 Medical College of Wisconsin, Milwaukee, WI USA; 3 Department of Obstetrics and Gynecology, Northwestern Medicine, Chicago, IL USA

**Keywords:** Complications, Hysterectomy, Sacrocolpopexy

## Abstract

**Introduction and Hypothesis:**

Minimally invasive sacrocolpopexy (SCP) with concomitant minimally invasive hysterectomy (MIH) is a frequently employed reconstructive surgical treatment for pelvic organ prolapse; however, the literature is limited regarding how the route of MIH affects postoperative adverse events. This study was aimed at investigating the association of route of MIH at the time of minimally invasive SCP and 30-day postoperative adverse events.

**Methods:**

This was a retrospective cohort study using the American College of Surgeons National Surgical Quality Improvement database to compare perioperative adverse events for patients who underwent minimally invasive SCP with MIH for uterovaginal prolapse. MIH included total laparoscopic hysterectomy (TLH), laparoscopic supracervical hysterectomy (SCH), laparoscopy-assisted vaginal hysterectomy (LAVH) and total vaginal hysterectomy (TVH). Perioperative adverse events of the four routes of MIH were compared.

**Results:**

During the study period, 7690 patients were identified. TLH was the most common route of MIH (56%), followed by laparoscopic SCH (39%), TVH (3%), and LAVH (2%). The rate of any 30-day adverse event was 10% and rates were similar between MIH groups. On multivariate logistic regression controlling for confounders, there were no significant differences in rates of postoperative adverse events between MIH groups.

**Conclusion:**

Adverse events following MIH at the time of SCP are not associated with the route of hysterectomy.

## Introduction

Pelvic organ prolapse (POP) is common, and the lifetime risk of surgery for POP is approximately 13% [1]. Several surgical options are available for the treatment of POP but sacrocolpopexy (SCP) is the gold-standard reconstructive treatment for apical prolapse owing to its superior durability compared with other options [2]. Prior studies have demonstrated lower rates of perioperative complications with minimally invasive SCP; therefore, SCP is now primarily performed using a minimally invasive approach [3–5]. In women with uterovaginal prolapse SCP most often involves a concomitant minimally invasive hysterectomy (MIH). MIH can be performed as a total laparoscopic hysterectomy (TLH), laparoscopic supracervical hysterectomy (SCH), laparoscopy-assisted vaginal hysterectomy (LAVH), and total vaginal hysterectomy (TVH).

Prior studies have shown low rates of postoperative complications after MIH performed for benign indications or at the time of SCP [6–9]. However, studies investigating differences in postoperative complications between different routes of MIH are limited [8, 9]. Therefore, the objective of this study was to determine the association between postoperative adverse events and route of MIH at the time of minimally invasive SCP for treatment of POP using a national surgical database. We hypothesized that there will be no difference in postoperative complications related to the route of MIH.

## Materials and Methods

We performed a retrospective cohort study using the 2019 to 2022 American College of Surgeons National Surgical Quality Improvement (ACS NSQIP) database. The study was considered exempt by the institutional review board. The ACS NSQIP database is nationally validated, risk adjusted, and outcome based, obtaining information directly from patient medical records rather than from insurance claims. There are more than 700 participating hospitals across the USA. The database collects preoperative risk factors, intraoperative variables, and 30-day outcomes for both inpatient and outpatient surgeries.

We identified women undergoing minimally invasive SCP with concurrent MIH for uterovaginal prolapse using Current Procedural Terminology codes. MIHs included TLH, laparoscopic SCH, LAVH, and TVH. We excluded patients whose American Society of Anesthesiology (ASA) class was not recorded, as well as patients who had missing variables for age, height, weight or operative time. Patients who had surgery for oncological indications were also excluded. Patient demographics, surgical characteristics, concomitant procedures, and postoperative data were extracted. Postoperative complications within 30 days of surgery were superficial incisional infection, organ space infection, wound disruption, pneumonia, unplanned intubation, pulmonary embolism, progressive renal insufficiency, urinary tract infection, stroke, cerebrovascular accident, ventilator requirement > 48 h, blood transfusions, venous thrombosis, sepsis, and septic shock.

The primary outcome of this study was to investigate the association of MIH route and any 30-day adverse event, which was defined as a composite outcome including any NSQIP-tracked postoperative complication, readmission, or reoperation. Secondary outcomes included differences in any 30-day postoperative complication, major complication, minor complication, reoperation, and readmission.

Statistical analysis was performed using R Studio Version 3.1. Differences in patient characteristics and outcomes across MIH approaches were described using Chi-squared test, Fisher’s exact test, and Student’s *t* test as appropriate. A multivariate logistic regression was performed to assess if MIH route was associated with any 30-day adverse event after controlling for any potential confounders. Variables that were statistically different on bivariate analysis with a *p* value < 0.05 and any variables that were considered clinically relevant were included in the multivariable logistic regression. We controlled for age, body mass index (BMI), current smoking status, hypertension, diabetes, concurrent midurethral sling, and ASA class. We also controlled for operative time by normalizing operative time for each route of MIH using z scores.

## Results

We identified 7690 patients who met the inclusion criteria. Mean age was 57 years (standard deviation [SD] ± 12) and mean BMI was 30 (SD ± 6). Most patients (80%) identified as white and most had an ASA class of 2 (73%). A total of 4319 (56%) underwent TLH, 2989 (39%) underwent SCH, 235 (3%) underwent TVH, and 147 (2%) had an LAVH. Concomitant procedures included anterior repair (3.1%), posterior repair (20.2%), combined anterior and posterior repair (9.1%), vaginal mesh insertion (0.2%), and paravaginal repair in 1 patient. Concomitant incontinence sling was placed during 37.5% of surgeries. Mean operative time was 180 min (SD ± 83). Between MIH groups there were statistically significant differences in age, race, BMI, hypertension, and smoking history (Table 1). Patients undergoing TLH were less likely to have an incontinence sling placed (*p* < 0.001). Operative time differed between groups, with TLH having the shortest operative time at 154 min (SD ± 78) and SCH having the longest operative time at 216 min (SD ± 77).

**Table 1:** Demographic and clinical characteristics of women undergoing sacrocolpopexy with minimally invasive hysterectomy

Variable	All (*N* = 7690)	TLH (*N* = 4319)	Laparoscopic SCH (*N* = 2989)	LAVH (*N* = 147)	TVH (*N* = 235)	*p* value
Age (years)^a^	56.8 ± 12.5	53.9 ± 12.9	60.8 ± 10.7	56.4 ± 13.1	58.9 ± 10.8	< 0.001
Race^b^						< 0.001
White	6168 (80.2)	3395 (78.6)	2461 (82.3)	121 (82.3)	191 (81.3)	
Asian	229 (3.0)	128 (3.0)	88 (2.9)	4 (2.7)	9 (3.8)	
Black	546 (7.1)	406 (9.4)	120 (4.0)	11 (7.5)	9 (3.8)	
Native American/Pacific Islander	23 (0.3)	12 (0.3)	10 (0.3)	0 (0.0)	1 (0.4)	
Unknown/other	724 (9.4)	378 (8.8)	310 (10.4)	11 (7.5)	25 (10.6)	
BMI (kg/m^2^)^a^	29.7 ± 6.1	30.5 ± 6.6	28.5 ± 5.2	29.6 ± 6.0	29.1 ± 5.2	< 0.001
Comorbidities^b^						
Diabetes	734 (9.5)	415 (9.6)	279 (9.3)	19 (12.9)	21 (8.9)	0.53
Hypertension	2424 (31.5)	1300 (30.1)	1001 (33.5)	48 (32.7)	75 (31.9)	0.02
Congestive heart failure	23 (0.3)	18 (0.4)	5 (0.2)	0 (0.0)	0 (0.0)	0.18
Renal failure	1 (0.0)	1 (0.0)	0 (0.0)	0 (0.0%)	0 (0.0)	0.84
Bleeding disorder	29 (0.4)	18 (0.4)	10 (0.3)	1 (0.7)	0 (0.0)	0.67
Smoking history^b^	647 (8.4)	471 (10.9)	146 (4.9)	13 (8.8)	17 (7.2)	< 0.001
ASA Class^b^						0.18
Class 1	544 (7.1)	302 (7.0)	217 (7.3)	14 (9.5)	11 (4.7)	
Class 2	5593 (72.7)	3134 (72.6)	2176 (72.8)	105 (71.4)	178 (75.7)	
Class 3	1531 (19.9)	874 (20.2)	585 (19.6)	26 (17.7)	46 (19.6)	
Class 4	22 (0.3)	9 (0.2)	11 (0.4)	2 (1.4)	0 (0.0)	
Concomitant procedures^b^						< 0.001
None	5180 (67.4)	3024 (70.0)	1925 (64.4)	93 (63.3)	138 (58.7)	
Anterior repair	237 (3.1)	115 (2.7)	90 (3.0)	5 (3.4)	27 (11.5)	
Posterior repair	1552 (20.2)	770 (17.8)	727 (24.3)	22 (15.0)	33 (14.0)	
Combined anterior and posterior repair	701 (9.1)	403 (9.3)	235 (7.9)	27 (18.4)	36 (15.3)	
Vaginal mesh insertion	19 (0.2)	6 (0.1)	12 (0.4)	0 (0.0)	1 (0.4)	
Paravaginal repair	1 (0.0)	1 (0.0)	0 (0.0)	0 (0.0)	0 (0.0)	
Concomitant sling^b^	2882 (37.5)	1320 (30.6)	1376 (46.0)	61 (41.5)	125 (53.2)	< 0.001
Operative time (minutes)^a^	180.1 ± 82.8	154.0 ± 77.7	216.3 ± 76.7	165.8 ± 68.7	210.3 ± 74.4	< 0.001
Length of stay (days)^a^	0.64 ± 2.7	0.62 ± 2.8	0.67 ± 2.8	0.67 ± 0.68	0.61 ± 0.55	0.89

^a^mean ± SD, ^b^*n* (%), *TLH* total laparoscopic hysterectomy, *SCH* supracervical hysterectomy, *LAVH* laparoscopy-assisted vaginal hysterectomy, *TVH* total vaginal hysterectomy, *BMI* body mass index, *ASA* American Society of Anesthesiologists

The rate of any 30-day adverse event was 10% and any 30-day postoperative complication was 8.7% (Table 2). Rates of any 30-day adverse event and any 30-day postoperative complication were similar in the two groups (*p* = 0.16 and 0.26 respectively). Laparoscopic SCH was associated with a lower rate of major complications at 1.4%, compared with 3.1% for TLH, 4.1% for LAVH, and 2.3% for TVH (*p* = 0.001). Rates of minor complications, readmission, and reoperations were similar in the two groups. The most common complication was a urinary tract infection (UTI), at 3.6% (Table 3). Rates of UTI were similar in the two groups (*p* = 0.49). Supracervical hysterectomy was associated with lower odds of organ/space surgical-site infections (0.3% compared with 1.0% for TLH, 2.0% for LAVH, and 0.9% for TVH; *p* = 0.003) and sepsis (0.1% compared with 0.3% for TLH, 2.0% for LAVH, and 0.9% for TVH; *p* < 0.001).

**Table 2:** Thirty-day adverse events in women undergoing sacrocolpopexy with minimally invasive hysterectomy

	All	TLH (*N* = 4319)	Laparoscopic SCH (*N* = 2989)	LAVH (*N* = 147)	TVH (*N* = 235)	*p* value
Any 30-day adverse event	580 (10.0)	336 (10.6)	213 (9.1)	16 (12.9)	15 (8.5)	0.16
Any 30-day complication	504 (8.7)	286 (9.0)	191 (8.2)	15 (12.1)	12 (6.8)	0.26
Major complication	138 (2.4)	95 (3.1)	34 (1.5)	5 (4.1)	4 (2.3)	0.001
Minor complication	393 (5.1)	207 (4.8)	166 (5.6)	10 (6.8)	10 (4.3)	0.34
Readmission	154 (2.0)	91 (2.1)	50 (1.7)	5 (3.4)	8 (3.4)	0.13
Reoperation	75 (1.0)	53 (1.2)	19 (0.6)	1 (0.7)	2 (0.9)	0.09

Data are *n* (%)

*TLH* total laparoscopic hysterectomy, *SCH* supracervical hysterectomy, *LAVH* laparoscopy-assisted vaginal hysterectomy, *TVH* total vaginal hysterectomy

**Table 3:** Postoperative complications

Postoperative complications	All (*N* = 7690)	TLH (*N* = 4319)	Laparoscopic SCH (*N* = 2989)	LAVH (*N* = 147)	TVH (*N* = 235)	*p* value
Superficial incisional SSI	123 (1.6)	68 (1.6)	52 (1.7)	3 (2.0)	0 (0.0)	0.22
Organ/space SSI	58 (0.8)	43 (1.0)	10 (0.3)	3 (2.0)	2 (0.9)	0.003
Wound disruption	6 (0.1)	4 (0.1)	2 (0.1)	0 (0.0)	0 (0.0)	0.93
Pneumonia	9 (0.1)	9 (0.2)	2 (0.1)	0 (0.0)	0 (0.0)	0.61
Unplanned intubation	3 (0.0)	2 (0.0)	1 (0.0)	0 (0.0)	0 (0.0)	0.97
Pulmonary embolism	16 (0.2)	11 (0.3)	4 (0.1)	0 (0.0)	1 (0.4)	0.55
Renal insufficiency	6 (0.1)	2 (0.1)	4 (0.1)	0 (0.0)	0 (0.0)	0.88
Urinary tract infection	275 (3.6)	143 (3.3)	115 (3.8)	7 (4.8)	10 (4.3)	0.49
Stroke/CVA	2 (0.0)	1 (0.0)	1 (0.0)	0 (0.0)	0 (0.0)	0.98
Ventilator requirement > 2 days	2 (0.0)	1 (0.0)	1 (0.0)	0 (0.0)	0 (0.0)	0.98
Blood transfusion	26 (0.3)	18 (0.4)	7 (0.2)	1 (0.7)	0 (0.0)	0.07
Venous thrombosis	11 (0.1)	5 (0.1)	5 (0.2)	0 (0.0)	1 (0.4)	0.56
Sepsis	24 (0.3)	15 (0.3)	4 (0.1)	3 (2.0)	2 (0.9)	< 0.001
Septic shock	7 (0.1)	3 (0.1)	4 (0.1)	0 (0.0)	0 (0.0)	0.76

Data are *n* (%)

*TLH* total laparoscopic hysterectomy, *SCH* supracervical hysterectomy, *LAVH* laparoscopy-assisted vaginal hysterectomy, *TVH* total vaginal hysterectomy

A multivariable logistic regression model was performed using statistically significant factors on bivariate analysis and those deemed clinically relevant. Final variables included age, BMI, current smoking status, concurrent midurethral sling, concurrent anterior repair, posterior repair or combined anterior and posterior repair, and operative time z score. After controlling for potential confounding factors, there were no differences in the rates of postoperative adverse events between MIH groups (Table 4). Concomitant midurethral sling was associated with increased odds of any 30-day adverse event (aOR 1.41; 95% CI 1.17–1.70).

**Table 4:** Multivariable logistic regression evaluating any 30-day adverse event

Variable	Adjusted odds ratio	95% confidence interval
Hysterectomy		
TLH^a^	–	–
SCH	0.94	0.78, 1.14
LAVH	1.28	0.72, 2.14
TVH	0.75	0.41, 1.27
Age	0.98	0.97, 0.99
BMI	1.01	1.00, 1.03
Operative time z score	1.34	1.23, 1.45
Sling	1.41	1.17, 1.70
Concomitant procedure		
Anterior repair	0.80	0.44, 1.36
Posterior repair	0.98	0.78, 1.22
Combined anterior and posterior repair	1.09	0.81, 1.45
Smoking history	1.27	0.93, 1.69

*TLH* total laparoscopic hysterectomy, *SCH* laparoscopic supracervical hysterectomy, *LAVH* laparoscopy-assisted vaginal hysterectomy, *TVH* total vaginal hysterectomy, *BMI* body mass index

^a^Reference

## Discussion

Our retrospective analysis of a large cohort of women undergoing prolapse repair with minimally invasive SCP and concurrent MIH found that the route of MIH was not associated with differences in rates of postoperative adverse events. The overall rate of 30-day adverse events was low at 10%. The rate of 30-day postoperative complications was 8.7%, and the most common complication was UTI at 3.6%. Rates of readmission and reoperation were also low at 2% and 1% respectively.

Initially, our univariate analysis found a statistically significant decrease in the rate of major complications, specifically sepsis and organ/space surgical-site infections; however, this association was not seen in the multivariable logistic regression. The baseline characteristics differed between groups and the difference in baseline characteristics was not accounted for in the univariate analysis. The multivariable logistic regression controlled for potential confounding by differences in baseline characteristics and the results suggest that the initial association was due to confounding rather than a true association based on the route of hysterectomy.

Several studies have investigated rates of postoperative complications after prolapse repair. The rate of postoperative complications reported in our study is similar to that reported in the literature [6, 9–11]. Rates of readmission and reoperation were also similar [8, 11, 12].

Our finding that route of MIH is not associated with differences in rates of postoperative adverse events is consistent with those of prior studies. A small retrospective study by Davidson et al. investigated differences in postoperative outcomes between women undergoing TVH and those undergoing laparoscopic SCH at the time of SCP [8]. Their study found that there were no differences in postoperative complications, readmission, or reoperations between the two routes of hysterectomy. Additionally, Cardenas-Trowers et al. also performed a study investigating postoperative complications after minimally invasive SCP and concurrent MIH using the NSQIP database; however, they used data from 2006–2015 [9]. They also found no differences in postoperative complications between TLH, laparoscopic SCH, TVH, and LAVH.

We found that concurrent midurethral sling placement was associated with an increased risk of complications. We hypothesize that this is due to an increased risk of voiding dysfunction after prolapse surgery with concurrent sling placement, which increases the risk of UTI [13]. UTI was the most common complication observed in our study and we suspect that this is the primary complication contributing to the association between sling placement and risk of complications.

Strengths of this study include the use of a reliable national database that systematically collects and maintains information on complications, readmissions, and reoperations. The data are collected from a large number of hospitals across the USA, which allows the findings of our study to be more generalizable than those of studies that include a single institution or a few institutions, as these can be influenced by regional bias. However, limitations of this study include its retrospective nature, which limits our ability to make inferences about causality. Another limitation is that the complications collected by NSQIP are not comprehensive of all possible postoperative complications and that complications, readmissions, and reoperations that occur after the 30-day window are not captured. Therefore, our study likely underestimates the rate of postoperative complications and is not reflective of long-term postoperative outcomes. One of these long-term outcomes is mesh exposure after sacrocolpopexy, a complication that is not tracked by NSQIP and therefore could not be analyzed in our study, but is an important complication that physicians and patients consider when choosing surgery. NSQIP does not track prolapse cures or recurrences and therefore this is an outcome that we were also unable to assess. Additionally, the NSQIP database does not distinguish between traditional laparoscopic surgeries and robotics-assisted surgeries, which did not allow us to compare complication rates for these modalities. Our study only included MIH that were performed concurrently with sacrocolpopexy and therefore the outcomes reported in our study may not be reflective of outcomes when hysterectomy is performed for other indications or concomitantly with other procedures.

Our study found that route of MIH is not associated with differences in postoperative adverse events. The overall rate of 30-day adverse events was low, at 10%, and the rate of 30-day complications was low as well, at 8.7%. Our findings suggest that surgeons could choose whichever route of MIH they prefer at the time of SCP, without concern for differences in rates of adverse events based on the route of MIH. 

## Data Availability

We do not own the data used in this study. The data are available by request from the American College of Surgeons.
